# Validity of a noninvasive estimation of deep body temperature when wearing personal protective equipment during exercise and recovery

**DOI:** 10.1186/s40779-019-0208-7

**Published:** 2019-06-14

**Authors:** Andrew P. Hunt, Mark J. Buller, Matthew J. Maley, Joseph T. Costello, Ian B. Stewart

**Affiliations:** 1 School of Exercise and Nutrition Sciences & Institute of Health and Biomedical Innovation, Queensland University of Technology, Brisbane, QLD Australia; 2 United States Army Research Institute of Environmental Medicine, Natick, MA USA; 3 Extreme Environments Laboratory, Department of Sport and Exercise Science, University of Portsmouth, Portsmouth, UK

**Keywords:** Protective clothing, Body core temperature, Heat strain, Heat stress, Kalman filter, Explosive ordnance disposal

## Abstract

**Background:**

Deep body temperature is a critical indicator of heat strain. However, direct measures are often invasive, costly, and difficult to implement in the field. This study assessed the agreement between deep body temperature estimated from heart rate and that measured directly during repeated work bouts while wearing explosive ordnance disposal (EOD) protective clothing and during recovery.

**Methods:**

Eight males completed three work and recovery periods across two separate days. Work consisted of treadmill walking on a 1% incline at 2.5, 4.0, or 5.5 km/h, in a random order, wearing EOD protective clothing. Ambient temperature and relative humidity were maintained at 24 °C and 50% [Wet bulb globe temperature (WBGT) (20.9 ± 1.2) °C] or 32 °C and 60% [WBGT (29.0 ± 0.2) °C] on the separate days, respectively. Heart rate and gastrointestinal temperature (T_GI_) were monitored continuously, and deep body temperature was also estimated from heart rate (ECTemp).

**Results:**

The overall systematic bias between T_GI_ and ECTemp was 0.01 °C with 95% limits of agreement (LoA) of ±0.64 °C and a root mean square error of 0.32 °C. The average error statistics among participants showed no significant differences in error between the exercise and recovery periods or the environmental conditions. At T_GI_ levels of (37.0–37.5) °C, (37.5–38.0) °C, (38.0–38.5) °C, and > 38.5 °C, the systematic bias and ± 95% LoA were (0.08 ± 0.58) °C, (− 0.02 ± 0.69) °C, (− 0.07 ± 0.63) °C, and (− 0.32 ± 0.56) °C, respectively.

**Conclusions:**

The findings demonstrate acceptable validity of the ECTemp up to 38.5 °C. Conducting work within an ECTemp limit of 38.4 °C, in conditions similar to the present study, would protect the majority of personnel from an excessive elevation in deep body temperature (> 39.0 °C).

## Background

Clothing worn by technicians during explosive ordnance disposal (EOD) is vital for protecting the wearer from ballistic threats during their work. However, the barrier it creates between the wearer and the environment, as well as the weight and bulk of the clothing, both impair body heat loss [1, 2] and increase metabolic rate [3], leading to a warmer deep body temperature. Consequently, EOD technicians experience elevated cardiovascular and thermoregulatory strains that impair their tolerance to work performed in the heat [1, 4–8]. Work duration limits are commonly employed to manage the risks of heat exhaustion and heat stroke and are based on expected elevations in deep body temperature of up to 38.0 °C or 38.5 °C for the average individual, with the assumption that individual variation will allow a small proportion of personnel to reach a maximum deep body temperature of 39.0 °C [9–12]. Therefore, an accurate measurement of deep body temperature is important for establishing work guidelines and for monitoring technicians during training and operations.

A range of measurement locations and devices are available for researchers and practitioners to monitor deep body temperature, each with its strengths and practical constraints. The most accurate locations for deep body temperature measurement are the esophagus or the rectum. However, the invasiveness of these measures often constrains their use to the laboratory [13]. Alternatively, the ingestion of temperature sensing pills provides a valid and field expedient, direct measure of deep body temperature [14, 15]. Although accurate, there are several practical constraints to their use. Specifically, they are financially costly to use regularly and may require a calibration check for optimal measurement accuracy [16, 17]. Furthermore, temperature variations along the gastrointestinal tract [13] and those resulting from food and fluid ingestion [18] can obscure a true deep body temperature reading at a given time. Aside from these invasive measurement techniques, a range of noninvasive measurement devices are available as surrogate measures of deep body temperature. Although temperatures such as oral, forehead, aural, and axilla are easily measured in the field, their validity has been questioned as they are not in close agreement with rectal temperature [14, 15, 19, 20]. As such, there are continued research efforts to develop a measure of deep body temperature that is both valid, field expedient, and noninvasive.

Recent advances have seen the development of techniques to estimate deep body temperature derived from noninvasive and field expedient measures. For example, measures of skin heat flux at several sites, heart rate, and skin temperature have been utilized to estimate deep body temperature during exercise in the heat, revealing a root mean square deviation (RMSD) of (0.28–0.34) °C [21]. Similar accuracy has been achieved with other models, such as utilizing heart rate and ambient temperature along with the input of individual height, weight, and clothing [RMSD (0.05–0.31) °C] [22], or chest skin temperature and load carried (RMSD 0.16 °C) [23], or heart rate, skin temperature, physical activity, ambient temperature, and relative humidity (RMSD 0.33 °C) [24]. A model has also been developed that utilizes insulated skin temperature, clothing microclimate temperature, heart rate and work to estimate rectal temperature, with a standard error of the estimate of 0.27 °C [25]. These methods have shown the potential to make accurate real-time monitoring of physiological strain a reality for managing the risks of work performed in the heat. However, they require multiple input parameters and therefore a range of sensor technologies to be available.

To date, only one method relies on a single noninvasive and commonly available physiological variable, namely, heart rate, to estimate deep body temperature [26]. Across a wide range of military populations and activities, the heart rate estimation of deep body temperature (ECTemp) has shown good agreement [RMSD 0.30 °C; systematic bias (− 0.03 ± 0.32) °C; 95% limits of agreement (LoA) ±0.63 °C] with either rectal temperature or gastrointestinal temperature [26]. Further studies among military populations have confirmed similar levels of agreement [RMSD 0.21 °C; systematic bias (0.02 ± 0.11) °C; 95% LoA ±0.48 °C] when conducting work wearing chemical and biological (CB) protective clothing [27], when wearing combat uniforms during jungle operations (− 0.01 °C; 95% LoA ±0.58 °C) [28], and during road marching (0.02 °C; 95% LoA ±0.76 °C) [23]. Slightly wider systematic bias has been observed during treadmill exercise wearing athletic clothing [(0.3 ± 0.4) °C; 95% LoA ± 0.7 °C) or CB protective clothing [(− 0.1 ± 0.4) °C; 95% LoA ±0.7 °C] [29]. These studies have shown that ECTemp, while not a replacement for direct measurement techniques, provides a noninvasive indication of deep body temperature that could easily be implemented in a range of athletic and occupational situations, such as technicians wearing EOD protective clothing.

While the research to date has examined the level of agreement during acute work bouts, or over extended periods of time, it has not specifically examined the level of agreement during repeated work and recovery periods. It has been demonstrated that during recovery from work performed in the heat, there are considerable adjustments to cardiovascular control and hemodynamic shifts that affect the heat loss mechanisms [30–32]. Consequently, deep body temperature can remain elevated during prolonged periods of recovery [32]. As such, it could be hypothesized that estimates of deep body temperature based on heart rate may pre-emptively estimate the return of deep body temperature to baseline, which could result in an increase in the error of estimation during recovery periods. This type of work with repeated bouts of exercise and recovery is routine for technicians wearing EOD protective clothing. Due to the severe restrictions imposed by EOD clothing on body heat loss, it is common practice for technicians to withdraw from the work area for rest periods. It is likely that technicians would work in pairs such that while one works, the other is resting. During rest periods, protective clothing is removed to facilitate heat loss. Therefore, this study aimed to assess the agreement between deep body temperature estimated from heart rate and that measured directly during repeated work bouts wearing EOD protective clothing and during recovery periods.

## Methods

### Participants

Eight healthy males [age: (26.4 ± 6.0) years; height: (1.8 ± 0.1) m; body mass: (77.4 ± 8.8) kg; peak aerobic capacity (V̇O_2peak_)_:_ (58.0 ± 5.2) mL/kg/min, the sum of eight skinfolds: (70.6 ± 25.1) mm] provided their informed and voluntary consent to participate in this study. All participants were recreationally active staff and students at the university. All tests and procedures in this study were approved by the University Human Research Ethics Committee (#1000001160).

The participants’ V̇O_2peak_ and body composition were assessed in a preliminary testing session. For the assessment of V̇O_2peak_ and maximal heart rate, participants were allowed a warm-up period on the treadmill, in which a comfortable running speed was determined for use during the maximal and incremental test. Participants then donned the expired gas analysis equipment (Moxus, AEI Technologies, Pennsylvania, USA) and a heart rate monitor (Polar Team^2^, Kempele, Finland) and stood on the treadmill for resting data collection. The test started at a speed of 4 km/h below the participants’ comfortable running speed, with a 1% grade. Every minute, the speed was increased by 1 km/h until the chosen speed was attained. Thereafter, the grade was increased by 1% every minute until volitional exhaustion. The variables used for determination of V̇O_2peak_ included a plateau in V̇O_2_ (i.e., < 150 ml/min change with an increase in workload); heart rate within 10 beats per minute of age predicted maximum (i.e., 220-age); respiratory exchange ratio > 1.10; and a rating of perceived exertion ≥19. Similar to previous investigations [33], where two criteria were met, the two highest successive 15 s values for V̇O_2_ defined the participants’ V̇O_2peak_.

Body composition was evaluated from subcutaneous skinfold thickness. Skinfold thickness was measured using Harpenden calipers (John Bull, West Sussex, UK) at eight sites (biceps, triceps, subscapular, iliac crest, supraspinale, abdomen, anterior thigh and medial calf). These sites were chosen to represent all body segments and were identified in accordance with the International Society for the Advancement of Kinanthropometry (ISAK) standards and were measured by an ISAK accredited anthropometrist [34].

Participants were familiarized with the EOD protective clothing. This involved the participant donning the protective clothing and walking on the treadmill at the speeds to be utilized for the trials.

### Research design

Participants were instructed to refrain from alcohol, tobacco, caffeine, and strenuous exercise and to consume 45 mL of water per kg of body mass in the 24 h before attending each of the work sessions. Participants were also requested to swallow an ingestible temperature sensor (CorTemp, HQ Inc., Palmetto, FL, USA) the evening prior, at least 8 h prior to the trials. This was to ensure that the optimal placement was achieved within the gastrointestinal tract and that the measurement was not influenced by food and fluid ingestion for the duration of the work and recovery bouts [18, 35, 36]. Upon presentation to the laboratory, euhydration status was confirmed by measuring urine specific gravity (USG, PAL 10s, ATAGO, Tokyo, Japan). Participants were required to produce a urine sample with USG ≤1.020 on each test day to confirm hydration status [37]. If participants did not meet the above guidelines, they were given an additional 500 ml of room temperature water to be consumed before the commencement of the trial.

Participants completed two test days (commencing between 8:30–10:00 am), and within each day, participants completed three work intensities for a total of six work periods. Work consisted of treadmill walking on a 1% incline at either 2.5, 4.0, or 5.5 km/h (corresponding to approximately 390, 550, and 795 watts of metabolic heat production [3]) for Moderate, Heavy and Very Heavy work intensities, respectively, until predefined end-point criteria were reached. The end-point criteria were in accordance with standardized guidelines [38] and included (1) gastrointestinal temperature (T_GI_) of > 39.0 °C; (2) heart rate 90% of maximum; (3) subjective fatigue or nausea; or (4) 60 min of exercise. Heart rate and T_GI_ data were recorded at 1 min intervals. Heart rate was measured throughout each test day using a heart rate monitor (Polar Team^2^, Kempele, Finland). Deep body temperature was measured using an ingestible pill administered the evening before the test day (CorTemp, HQ Inc., Palmetto, FL, USA). Work bouts were conducted in an environmental chamber that maintained the ambient temperature and relative humidity at either 24 °C and 50% [Temperate; Wet Bulb Globe Temperature (WBGT) (20.9 ± 1.2) °C] or 32 °C and 60% [Warm-wet; WBGT (29 ± 0.2) °C]. Wind speed was controlled at 4.7 km/h in all conditions. Only one environmental condition was completed on each test day, with test days separated by at least one week. The order of the work intensities within a day and the presentation of environmental conditions between days were randomized. The order of work intensities was randomized to simulate a work day for EOD technicians. During real work conditions, there is no set order to work demands, so the study was designed to reflect the potential for variability in their work.

The participants wore the Med-Eng™ EOD9 ensemble (Allen Vanguard, Ogdensburg, New York, USA) during each work period. The ensemble consisted of a jacket and trousers incorporating ballistic protection, groin protection, and a helmet (total mass: 33.4 kg). The garments were laundered in accordance with the manufacturer’s instructions in between each trial. In addition to the EOD suit, a chemical protective undergarment and respirator (Promask with a pro2000 PF10 filter; Scott Safety, Lancashire, UK) were also worn by the participants. These undergarments were either an Allen Vanguard (Explosive Protective Equipment, Newstead QLD 4006 Australia; 2.9 kg) (*n* = 6) or the Saratoga™ Hammer Suit (Applied Response Solutions, Georgetown, TX, USA; 4.2 kg) (*n* = 2). Both undergarments were air permeable and charcoal impregnated and comprised a jacket, trousers, booties, gloves, and hood. Participants wore the same undergarment on both days of testing. Participants wore a base ensemble underneath the protective clothing, which consisted of a T-shirt, shorts, socks, underwear, and athletic shoes with a soft rubber sole. These base ensemble requirements were standardized in accordance with the American Society for Testing and Materials standards for the determination of physiological responses of the wearer to protective clothing ensembles, ASTM F2668–07 [38]. All garments were laundered in between each trial.

Between exercise periods, participants removed all protective clothing and rested (seated) in an air-conditioned laboratory wearing only the base ensemble. Participants were allowed to consume food and fluid ad libitum during recovery. Participants could choose from water or sports drink with electrolytes for fluid replenishment. Food options included sandwiches, muesli bars, and fruit. Deep body temperature and heart rate were continuously monitored, and following their return to baseline levels (T_GI_ ± 0.5 °C; HR ±10%), the participant commenced donning the protective clothing for the subsequent exercise period. After the final work period, the recovery period lasted until the participant’s heart rate returned below 100 beats/min.

### Deep body temperature estimation

Minute-to-minute deep body temperature was estimated (ECTtemp) from sequential measures of heart rate [26, 27]. The algorithm uses an extended Kalman Filter [39, 40] to generate time-stepped predictions of deep body temperature, which are then corrected by measurements of heart rate and the previously established relationship between heart rate and deep body temperature [26]. The inputs to the model included the participant’s baseline T_GI_ measured at the beginning of the test day, an assumed initial variance of zero, and their heart rate at one-minute intervals. Thereafter, at each one minute time interval, an iterative approach utilizing six equations (eqs. 1–6) was used to calculate the new estimate of deep body temperature (ECTemp) and its associated variance (v_t_) from the current HR observation (HR_t_), the previous minute deep body temperature estimate (CT_t-1_) and the previous variance (v_t-1_). To compute an ECTemp estimate of deep body temperature at any minute, compute each equation in order (eqs. 1 through 6). The results of eqs. 5 and 6 are saved and used in the next minute’s computations. Only a new observation of HR is needed to continue estimating deep body temperature over subsequent minutes. To start either set an initial *ECTemp*_*t-1*_ (e.g., a resting deep body temperature of 37.0 °C) and set *v*_*t-1*_ = 0.01; or set *ECTemp*_*t-1*_ = a measured deep body temperature and set *v*_*t-1*_ = 0). Note that the subscript (*t*) refers to a minute time step, either the current (*t*) or previous (*t-1*).

Equation 1: A preliminary estimate of deep body temperature (ĈT_t_) based on previous deep body temperature estimate (CT_t-1_)

$$ \hat{C}{T}_t={ECTemp}_{t-1} $$

Equation 2: A preliminary estimate of the variance (v̂_t_) of the deep body temperature estimate (CT_t_) based on the previous deep body temperature variance (v_t-1_)

$$ {\hat{v}}_t={v}_{t-1}+0.000484 $$

Equation 3: The Kalman filter mapping function variance component (c_t_)

$$ {c}_t=-9.1428\times \hat{C}{T}_t+384.4286 $$

Equation 4: The Kalman gain weighting factor (k_t_) based on the preliminary estimate of variance (v̂_t_) and the KF variance component (c_t_)

$$ {k}_t=\frac{{\hat{v}}_t{c}_t}{c_t^2{\hat{v}}_t+356.4544} $$

Equation 5: Compute this minutes estimate of deep body temperature (ECTemp_t_) using the preliminary time update estimate (ĈT_t_) and the Kalman gain (k_t_) weighting of the error between the HR_t_ observation and the expected HR (computed from the Kalman filter expected HR model in the inner parentheses of eq. 5 from the preliminary estimate of deep body temperature computed in eq. 1)

$$ {ECTemp}_t=\hat{C}{T}_t+{k}_t\left({HR}_t-\left(-4.5714\times \hat{C}{T}_t^2+384.4286\times \hat{C}{T}_t-7887.1\right)\right) $$

Equation 6: Compute the variance of this minutes deep body temperature estimate (v_t_)

$$ {v}_t=\left(1-{k}_t{c}_t\right)\ {\hat{v}}_t $$

### Agreement analysis

The agreement between T_GI_ and ECTemp was evaluated by systematic bias and 95% limits of agreement (95% LoA), root mean squared error (RMSE), mean absolute error (MAE), and Lin’s concordance coefficient (Pc) [41]. Limits of agreement were calculated with a modified standard deviation to account for the repeated measures in study participants, as described by Bland and Altman [42]. These statistics were calculated for all of the data from all participants combined and separated into exercise and recovery periods and were plotted visually with scatterplots, Bland-Altman plots, and frequency distributions of the error. Systematic bias, limits of agreement, RMSE, MAE, and Lin’s concordance were also calculated for each individual over a whole test day and for separate exercise and recovery periods. In addition, systematic bias and limits of agreement were also calculated for levels of T_GI_, including (37.0–37.5) °C, (37.5–38.0) °C, (38.0–38.5) °C, and > 38.5 °C. For ECTemp to be considered a clinical grade measure of deep body temperature, it was required to show a systematic bias < 0.1 °C [35]. Alternatively, for ECTemp to be considered suitable for field applications, systematic bias should be < 0.27 °C [14, 15]. Furthermore, consideration was given to the variation in the error of prediction, such that limits of agreement should be within ±0.40 °C for acceptance in clinical grade [35] or ± 0.7 °C for acceptance in field applications [43, 44]. The allowance of these variations corresponds to the expected spatial variation in deep body temperature between different locations of measurement, such as rectal and esophageal [13]. The variation in predictive accuracy, standard error of the estimate, was used to evaluate the maximum predicted deep body temperature that would be permissible in a workplace setting to ensure that the majority of individuals would not exceed a deep body temperature of 39.0 °C.

### Statistical tests

Repeated measures ANOVA (IBM SPSS Statistics, v23) assessed for differences in agreement between the environmental conditions and the exercise and recovery periods [1–3]. Statistical significance was accepted at α < 0.05.

## Results

**Fig. 1:**
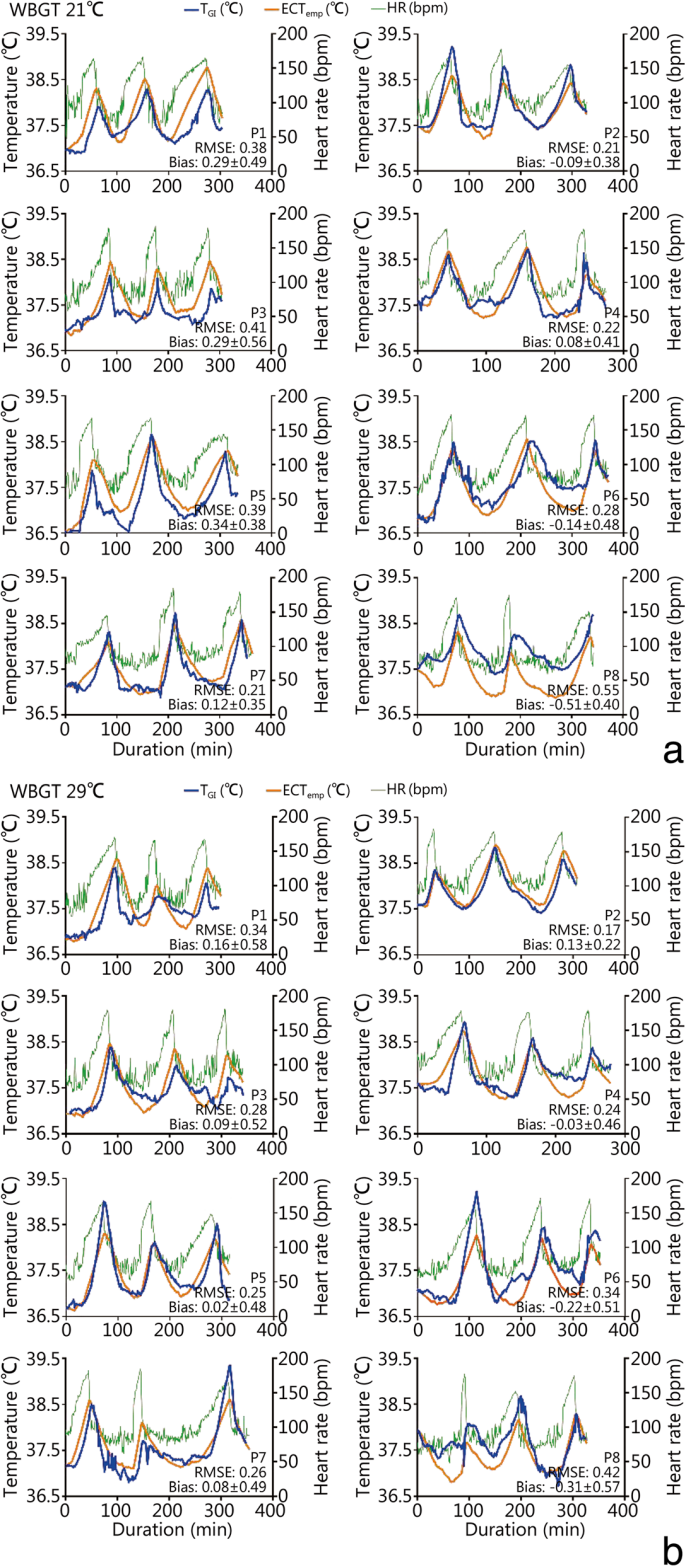
Individual traces of T_GI_ (blue line), ECTemp (orange line), and heart rate (green line) in WBGT 21 °C (**a**) and 29 °C (**b**) conditions. P1-P8 represent each participant, 1 to 8. RMSE: Root mean squared error; ECTemp: Estimated core temperature; WBGT: Wet bulb globe temperature

Individual traces of T_GI_, ECTemp and HR throughout the day are provided in Fig. 1. In the temperate conditions, the work durations were (39 ± 15) min, (33 ± 14) min and (40 ± 18) min, and the recovery periods were (78 ± 16) min, (85 ± 18) min, and (29 ± 5) min following work periods 1, 2, and 3, respectively. Work durations for categories of work intensity included (54 ± 7) min, (36 ± 7) min, and (21 ± 6) min for moderate, heavy, and very heavy work categories, respectively. In the warm-wet condition, work durations were on average (34 ± 16) min, (28 ± 12) min, and (30 ± 15) min, and recovery times following work periods were (77 ± 11) min, (84 ± 15) min, and (31 ± 7) min. Work durations for categories of work intensity included (44 ± 9) min, (32 ± 7) min, and (16 ± 5) min for moderate, heavy, and very heavy work categories, respectively. The majority of work periods (79%) were terminated due to reaching the heart rate end-point criteria. Four trials (8%) were terminated due to reaching a T_GI_ of 39.0 °C; two occurred during moderate work in warm-wet conditions, one during moderate work and one during heavy work in temperate conditions. Three (6%) were terminated after 60 min of work, all of which were during the moderate work intensity in temperate conditions. Three work periods were terminated due to volitional fatigue, two during moderate work in warm-wet, and one at moderate intensity in temperate conditions.

**Fig. 2:**
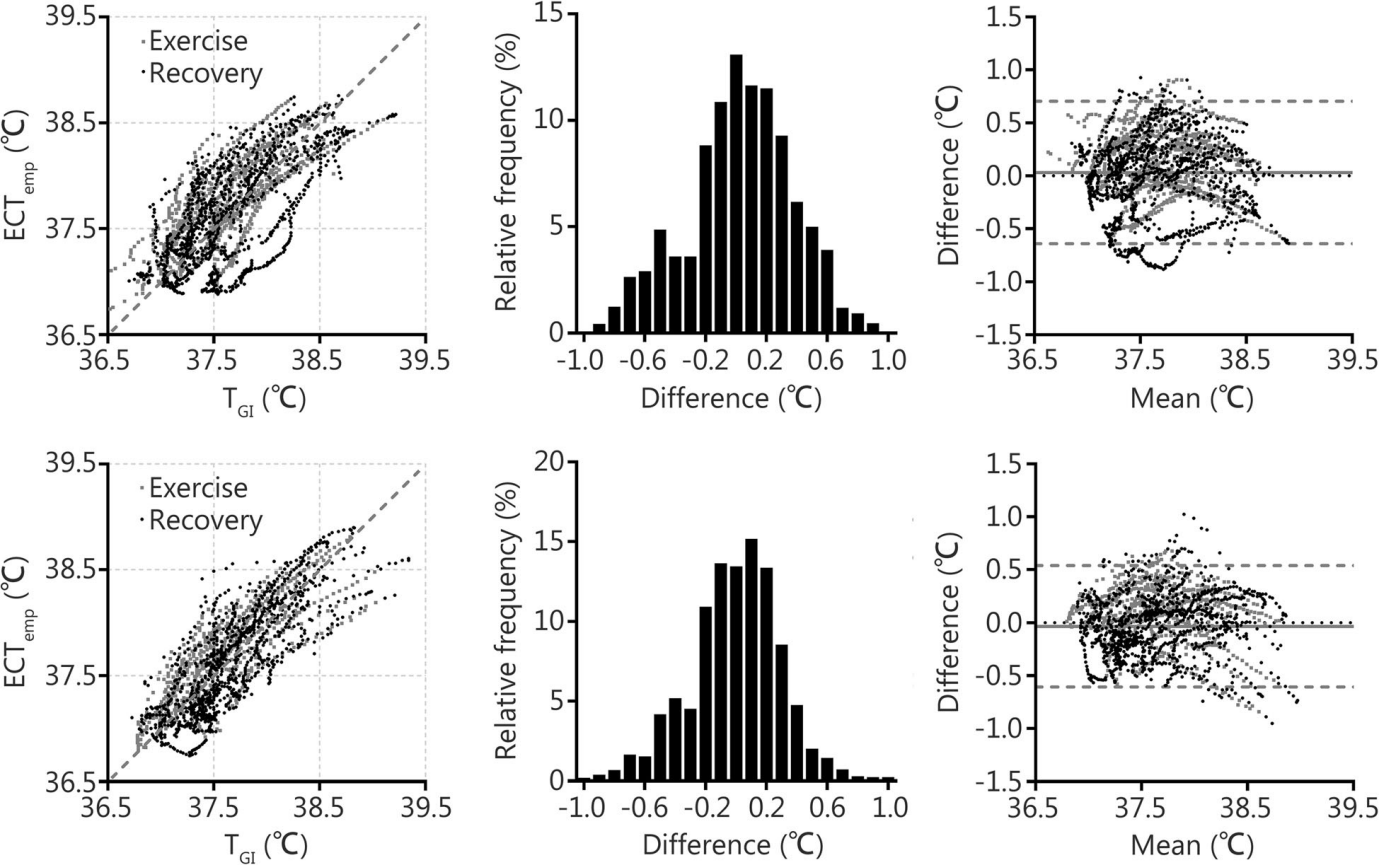
Scatterplot (left), frequency distribution (middle), and Bland-Altman plot (right) of T_GI_ and ECTemp throughout both exercise and recovery periods in conditions of WBGT 21 °C (Top) and 29 °C (Bottom). The difference was calculated as ECTemp minus T_GI_

Analysis of the agreement between T_GI_ and ECTemp revealed that the systematic bias was 0.01 °C with a 95% LoA of ±0.64 °C and the standard error of the estimate (SEE) was ±0.3 °C when all data were combined across all participants, environmental conditions, and work/recovery periods (Table 1, Fig. 2). During exercise periods, ECTemp overestimated T_GI_ by a systematic bias of (0.10–0.13) °C (Table 1). Alternatively, during recovery periods, ECTemp slightly underestimated T_GI_ by a systematic bias of − 0.04 °C (Table 1). At T_GI_ levels of (37.0–37.5) °C, (37.5–38.0) °C, (38.0–38.5) °C, and > 38.5 °C, systematic bias and ± 95% LoA were (0.08 ± 0.58) °C, (− 0.02 ± 0.69) °C, (− 0.07 ± 0.63) °C, and (− 0.32 ± 0.56) °C, respectively. This level of variation in prediction shows that to ensure that the majority of individuals (97.5%) have a deep body temperature of less than 39.0 °C, work should cease at an ECTemp reading of 38.4 °C (calculated as 39.0 °C minus two-times the standard error of estimate). Furthermore, a sensitivity of 86% was observed, correctly classifying 19 out of 22 T_GI_ data points that were above 39.0 °C, based on an ECTemp greater than 38.4 °C.

**Table 1:** Agreement statistics between T_GI_ and ECTemp for exercise, recovery, and combined periods in 21 °C and 29 °C environmental conditions

Item	Bias (°C)	LoA (°C)	RMSE (°C)	MAE (°C)	Pc
WBGT 21					
Ex	0.13	±0.60	0.32	0.26 ± 0.18	0.73
Rec	−0.04	±0.78	0.38	0.30 ± 0.23	0.69
All	0.03	±0.70	0.35	0.27 ± 0.21	0.74
WBGT 29					
Ex	0.10	±0.53	0.28	0.23 ± 0.16	0.81
Rec	−0.04	±0.58	0.29	0.23 ± 0.18	0.82
All	−0.00	±0.57	0.29	0.23 ± 0.18	0.82
Combined					
Ex	0.12	±0.57	0.30	0.25 ± 0.18	0.76
Rec	−0.04	±0.68	0.34	0.27 ± 0.21	0.76
All	0.01	±0.64	0.32	0.25 ± 0.20	0.78

*Bias* Systematic bias (mean difference: ECTemp minus T_GI_); *LoA* 95% limits of agreement, *RMSE* Root mean square error, *MAE* Mean absolute error, *Pc* Lin’s concordance correlation coefficient, *WBGT* Wet bulb globe temperature, *Ex* Exercise period, *Rec* Recovery period

The error statistics were also examined for each individual over the course of the test day (Fig. 1). Collectively, the average error statistics among participants showed that there were no significant differences in error between the exercise and recovery periods or the two environmental conditions (Table 2).

**Table 2:** Mean (±SD) of the error statistics from each individual for both WBGT conditions and across all work and recovery periods (°C, *n* = 8)

Item	Ex 1	R 1	Ex 2	R 2	Ex 3	R 3	Interaction	
							***F***	***P***
Systematic bias							0.15	0.979
WBGT 21	0.09 ± 0.27	0.01 ± 0.31	0.12 ± 0.21	−0.01 ± 0.37	0.15 ± 0.34	0.16 ± 0.35		
WBGT 29	0.07 ± 0.28	−0.04 ± 0.23	0.07 ± 0.22	− 0.11 ± 0.17	0.11 ± 0.20	0.07 ± 0.34		
LoA							1.23	0.316
WBGT 21	0.23 ± 0.07	0.39 ± 0.13	0.30 ± 0.11	0.32 ± 0.07	0.28 ± 0.10	0.30 ± 0.07		
WBGT 29	0.37 ± 0.21	0.40 ± 0.23	0.22 ± 0.09	0.30 ± 0.13	0.24 ± 0.11	0.31 ± 0.23		
RMSE							1.51	0.213
WBGT 21	0.26 ± 0.14	0.33 ± 0.15	0.26 ± 0.11	0.34 ± 0.19	0.34 ± 0.18	0.36 ± 0.19		
WBGT 29	0.33 ± 0.11	0.29 ± 0.14	0.22 ± 0.12	0.24 ± 0.08	0.23 ± 0.11	0.36 ± 0.13		
MAE							1.179	0.339
WBGT 21	0.24 ± 0.14	0.29 ± 0.15	0.23 ± 0.11	0.30 ± 0.20	0.31 ± 0.19	0.33 ± 0.19		
WBGT 29	0.29 ± 0.10	0.25 ± 0.12	0.20 ± 0.12	0.21 ± 0.08	0.21 ± 0.12	0.33 ± 0.14		

*Ex* Exercise period, *R* Recovery period, *LoA* 95% limits of agreement, *RMSE* Root mean square error, *MAE* Mean absolute error, *WBGT* Wet bulb globe temperature

## Discussion

This study aimed to assess the agreement between deep body temperature estimated from heart rate (ECTemp) and that measured directly during repeated work bouts wearing EOD protective clothing and during recovery periods. The present findings have confirmed that the systematic bias of the predicted deep body temperature falls between − 0.04 °C and 0.13 °C across work and recovery periods for personnel wearing EOD protective clothing (Table 1). However, the variation in error was in the order of ±0.64 °C (95% LoA). For this reason, the present technique does not meet the clinical grade agreement requirements to replace the direct criterion measurement of deep body temperature. On the other hand, the observed level of agreement was within the criteria set for monitoring deep body temperature in a field setting and was in accordance with the assumptions underpinning current occupational guidelines. In this context, the study findings demonstrate acceptable validity of the ECTemp prediction of deep body temperature up to 38.5 °C. Conducting work in hot environments within an ECTemp limit of 38.4 °C, in conditions similar to the present study, would protect the majority of individuals from an excessive elevation in deep body temperature (> 39.0 °C). However, further development and validation of the technique is required to improve the accuracy of predicting elevations in deep body temperature in excess of 38.5 °C.

Overall, the agreement between the measured and the predicted deep body temperature found in the present study is in close alignment with previous reports [23, 26–28]. Importantly, systematic bias centered closely around zero, both during exercise and recovery periods (Table 1). This degree of systematic bias is within the ±0.27 °C previously utilized in establishing the validity of temperature measurement devices for monitoring heat strain among athletes [14, 15] and is within the more conservative requirement of ±0.1 °C recommended when assessing the validity of gastrointestinal temperature against rectal and esophageal temperatures [35]. However, the variation in error must also be given careful consideration. The observed mean absolute error reveals that the average error is ±0.25 °C (Table 1). Since the error appears normally distributed (Fig. 2, middle panel), we can infer that approximately 50% (the average or less) of measurements will fall within ±0.25 °C of the measured deep body temperature. Furthermore, the 95% LoA showed that up to 95% of measurements fall within ±0.64 °C (Table 1). These limits of agreement are wider than the ±0.4 °C recommended for agreement with the direct criterion measures of deep body temperature such as rectal, esophageal, and gastrointestinal [35]. Consequently, it must be concluded that the ECTemp method is not a suitable replacement for these criterion measures of deep body temperature if a clinical grade measurement is required.

While the present technique did not meet the agreement requirements to replace the direct criterion measures, the findings demonstrated that agreement was within the criteria set for monitoring deep body temperature in a field setting. As a result, there is potential for the ECTemp to provide a noninvasive indication of deep body temperature that could be used in conjunction with occupational guidance limits to enhance risk management strategies for work performed in hot environments. Common workplace guidelines for managing the risk of heat-related injury place limits on the work duration to manage the risks of heat exhaustion and heat stroke. These limits are based on expected elevations in deep body temperature of up to 38.0 °C for general occupational/industrial limits or 38.5 °C in military settings [9–12]. With individual variations, these limits assume that 95% of personnel will experience deep body temperature responses within ±0.6 °C such that a small proportion of personnel may exhibit a deep body temperature up to 38.6 °C or 39.1 °C. When examining the accuracy of ECTemp at specific levels of T_GI_, up to 38.0 °C T_GI_, the systematic bias and 95% limits of agreement were (0.02 ± 0.69) °C, and up to 38.5 °C, they were (0.07 ± 0.63) °C. Furthermore, the results showed that a limit to ECTemp of 38.4 °C would protect most people (97.5%) from exceeding an actual deep body temperature of 39.0 °C, similar to previous estimation techniques [25]. Therefore, the ECTemp up to 38.4 °C provides a safety margin equivalent to the present workplace guidance limits. The potential advantage of using individual real-time predictions of deep body temperature is that those individuals who respond in the lower half of the population may work for extended periods while protection is still maintained for those with higher elevations in deep body temperature.

While the present study demonstrated acceptable agreement between deep body temperature and ECTemp in the range of deep body temperature associated with occupational tolerance limits of up to 38.0–38.5 °C [9–12], an important avenue for further examination of ECTemp should investigate higher peaks in deep body temperature. While most exercise trials in the present study ceased with deep body temperature in the range of 38.0–38.5 °C, several reached 39.0 °C. When agreement between T_GI_ and ECTemp was evaluated at varying levels of T_GI_, the systematic bias was found to be greater (− 0.32 °C) when T_GI_ was above 38.5 °C. On four occasions when the deep body temperature reached a peak between 38.8–39.1 °C, the ECTemp value underestimated the peak exercise value by 0.5–0.8 °C (Fig. 1: WBGT 21: P2 Ex 1; WBGT 30: P5 Ex 1, P6 Ex 1, P7 Ex 3). Future studies should examine similar elevations in deep body temperature to refine and validate the heart rate prediction of deep body temperature during intermittent work.

A novel finding of the present study was that the agreement between the measured deep body temperature and ECTemp was similar between exercise and recovery periods, as well as between the environmental conditions (Table 2). The success of the ECTemp is grounded in how changes in heart rate reflect the cumulative effects of heat stress on the thermoregulatory strain in the body. These include 1) the increase in heart rate observed due to both the metabolic cost of physical work and heat stress; 2) the cardiovascular adjustments responsible for the redistribution of blood flow and the maintenance of blood pressure in response to fluctuations in deep body and skin temperatures; 3) the core to skin temperature gradient [45–47]; and 4) the effects of dehydration on blood volume and cardiovascular strain [48]. Furthermore, it has been demonstrated that during recovery from work performed in the heat, there are considerable adjustments to cardiovascular control and hemodynamic shifts that affect the heat loss mechanisms [30–32]. Consequently, deep body temperature can remain elevated for prolonged periods of recovery [32]. Despite these physiological phenomena, ECTemp was consistent throughout the transitions between work and recovery in the present study. The physiology that underpins the ECTemp algorithm assumes that these thermoregulatory processes are reflected in heart rate. The success of the algorithm to accurately reflect deep body temperature during exercise and through recovery periods is supported by these underlying physiological mechanisms.

The ability to monitor deep body temperature in the field via noninvasive methods is a considerable advantage for managing the risk of heat-related injury in occupational settings [49, 50]. Knowledge of an individual approaching an excessive elevation in deep body temperature can be used to inform tolerance limits for work periods. Alternatively, confirmation that a sufficient physiological recovery has occurred before recommencing work may also aid in preventing the accumulation of excessive heat strain over the course of a workday. For EOD technicians, careful management of work and recovery periods is crucial to preventing heat-related injury [1, 4–7]. The implementation of wearable physiological monitoring technologies has been promoted as a way forward in enhancing workforce health during occupational heat stress [11, 49, 50]. Therefore, the approach of monitoring an easily measured physiological variable for the estimation of an important (all be it difficult to measure) indicator of heat strain (i.e., deep body temperature) may provide a balance between a validated measurement technique and practical application in occupational settings. To that end, recent research has pursued the development of real-time deep body temperature estimators. A range of models have been developed [21–25], each incorporating multiple parameters and showing acceptable agreement with measured deep body temperatures (RMSD’s of 0.50–0.33 °C). These approaches have the potential to provide low-cost, noninvasive, practical indications of heat strain in occupational settings. However, an advantage of the ECTemp is that while it produces a similar level of agreement with measured deep body temperature, it only utilizes a single input parameter, namely, heart rate.

As a potential field monitoring technique, ECTemp has shown closer agreement to the criterion measure of deep body temperature than other indirect measurement locations. A range of noninvasive and field expedient temperature measurement sites have shown unacceptably high error margins compared to rectal temperature. During indoor exercise and recovery, forehead temperature (bias: 0.29 °C; 95% LoA: ±2.27 °C), oral temperature (bias: -0.86 °C; 95% LoA: ±1.24 °C), aural temperature (bias: -0.67 °C; 95% LoA: ±1.02 °C), and axillary temperature (bias: -0.94 °C; 95% LoA: ±1.59 °C) all exhibited unacceptably high systematic bias (> 0.27 °C) and wide limits of agreement [15]. Similarly, measurement error during outdoor exercise and recovery in the field was unacceptably high for forehead temperature (bias: 0.60 °C; 95% LoA: ±1.70 °C), oral temperature (bias: -1.20 °C; 95% LoA: ±1.71 °C), aural temperature (bias: -1.00 °C; 95% LoA: ±1.14 °C), and axillary temperature (bias: -2.58 °C; 95% LoA: ±1.71 °C) than that of rectal temperature [14]. In contrast, the present study and others [26–28] demonstrated that the systematic bias of ECTemp is within the recommended ±0.27 °C [15, 37] and that the limits of agreement are much tighter than other available measurement techniques for field applications (Table 1). Therefore, in situations where direct measures of rectal, esophageal, or gastrointestinal temperature are not suitable, ECTemp appears to be a more accurate noninvasive indicator of deep body temperature during exercise and recovery than forehead, oral, aural or axillary temperature. However, further investigations to directly compare these indices are warranted.

Several limitations of the present study should be noted, such as the generalizability of the study findings, individual variations, and non-thermal effects on heart rate. While the present study was designed to simulate work intensity categories and metabolic heat productions likely to be experienced by EOD technicians in the field, it is possible that actual work activities and rest periods may vary from the present study. For example, EOD activities may involve intermittent work and varied body postures. Therefore, further study should investigate the validity of the ECTemp in an EOD field training environment. In addition, individual variation in aerobic capacity, body composition, age, and sex should be investigated to ensure a wider application to the general population. Finally, while thermal strain has a strong influence on heart rate, other non-thermal factors may also affect heart rate. Such factors include psychological stressors, circadian rhythm, and static exertion. These factors may influence the prediction of deep body temperature from heart rate and should be investigated further.

## Conclusions

In conclusion, the present study has confirmed that the systematic bias of ECTemp falls between − 0.04 and 0.13 °C across work and recovery periods for personnel wearing EOD protective clothing. While the variation in the error of prediction (95% LoA ±0.64 °C) is wider than is acceptable for a direct measure of deep body temperature, the observed level of agreement was within the criteria set for monitoring deep body temperature in a field setting. Performing work in hot environments within an ECTemp limit of 38.4 °C, in conditions similar to the present study, would protect the majority of individuals from an excessive elevation in deep body temperature (> 39.0 °C). However, further development and validation of the technique is required to improve the accuracy of predicting elevations in deep body temperature in excess of 38.5 °C.

## Data Availability

The datasets used and/or analyzed during the current study are available from the corresponding author upon reasonable request.

## Abbreviations

ECTemp: Estimated core temperature
EOD: Explosive ordnance disposal
LoA: Limits of agreement
MAE: Mean absolute error
RMSE: Root mean square error
T_GI_: Gastrointestinal temperature
V̇O_2peak_: Peak Aerobic Capacity
WBGT: Wet Bulb Globe Temperature.
